# Supplementary material for: Turning a native or corroded Mg alloy surface into an anti-corrosion coating in excited CO2
Source: Nat Commun. 2018 Oct 3;9:4058. doi: 10.1038/s41467-018-06433-5 (PMC6170486; doi:10.1038/s41467-018-06433-5)
Supplement: Supplementary file 1 — Supplementary Information [file 41467_2018_6433_MOESM1_ESM.pdf]

Supplementary Information

for

Turning native or corroded Mg alloy surface into anti-corrosion coating in excited CO_2

Wang et al.

Supplementary Figures

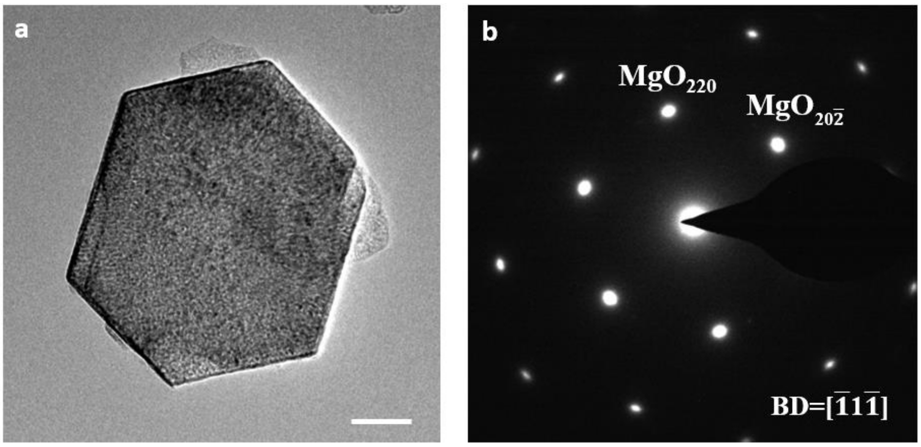

Supplementary Figure 1 | TEM characterizations of the single-crystalline MgO lamella before exposure to CO_2. (a) TEM image of a single flake of MgO crystal with hexagonal morphology. **(b)** Electron diffraction pattern of the MgO in (a) shows that each lamella is single crystalline. Beam direction (BD) is $[\bar{1}1\bar{1}]$. Scale bar, 50 nm.

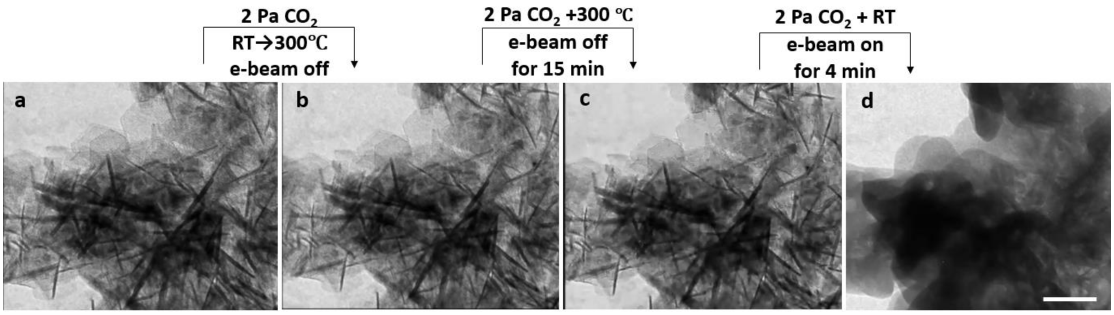

Supplementary Figure 2 | Comparison of the heating and e-beam irradiation effects on the chemical reaction, $MgO+CO_2=MgCO_3$. (a) TEM image of MgO flakes before exposure in CO_2. **(b)** Unchanged in 2 Pa CO_2 atmosphere without e-beam irradiation when the temperature increased from room temperature (RT) to 300°C. **(c)** The sample was held at 300°C for 15 min without e-beam irradiation, and it still kept the same morphology. After the sample was cooled down to RT and e-beam was on, MgO began to react with CO_2. **(d)** The morphology of product after exposure to excited CO_2^* after 4 min. Scale bar, 100 nm.

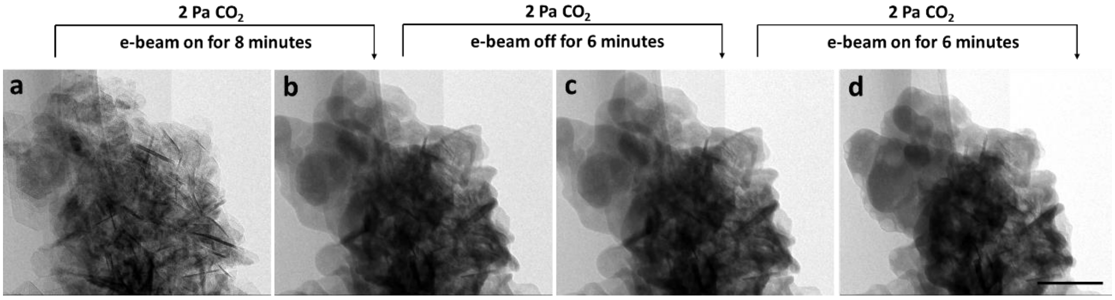

Supplementary Figure 3 | The reaction of MgO with excited CO_2^* shows no obvious "memory" effect for the prior electron beam irradiation. **(a)** The TEM bright field image of pristine MgO flakes. **(b)** The morphology of products after exposure in 2 Pa CO_2 with the e-beam irradiation for 8 minutes. **(c)** The products kept unchanged in 2 Pa CO_2 with the e-beam off for 6 minutes, indicating that prior e-beam irradiation effect disappears as soon as e-beam is discontinued. The reaction restarted again as soon as the e-beam was turned on. **(d)** The morphology of products after exposure in 2 Pa CO_2 with the e-beam irradiation for additional 6 minutes. Scale bar, 200 nm.

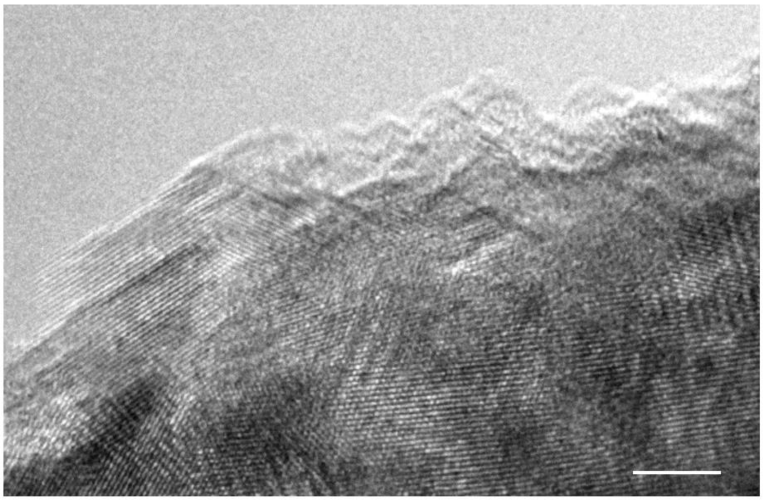

Supplementary Figure 4 | High-resolution TEM image of the surface layer after reaction. This is enlarged from the framed zone in Figure 2h. Different from the

reaction of excited CO_2 with pure MgO, the reaction with Mg metal underneath is moderate because of the reduced specific surface area of MgO that can be used for absorbing CO_2. So, the slowly generated $MgCO_3$ has sufficient time to crystallize rather than forming amorphous. Scale bar, 10 nm.

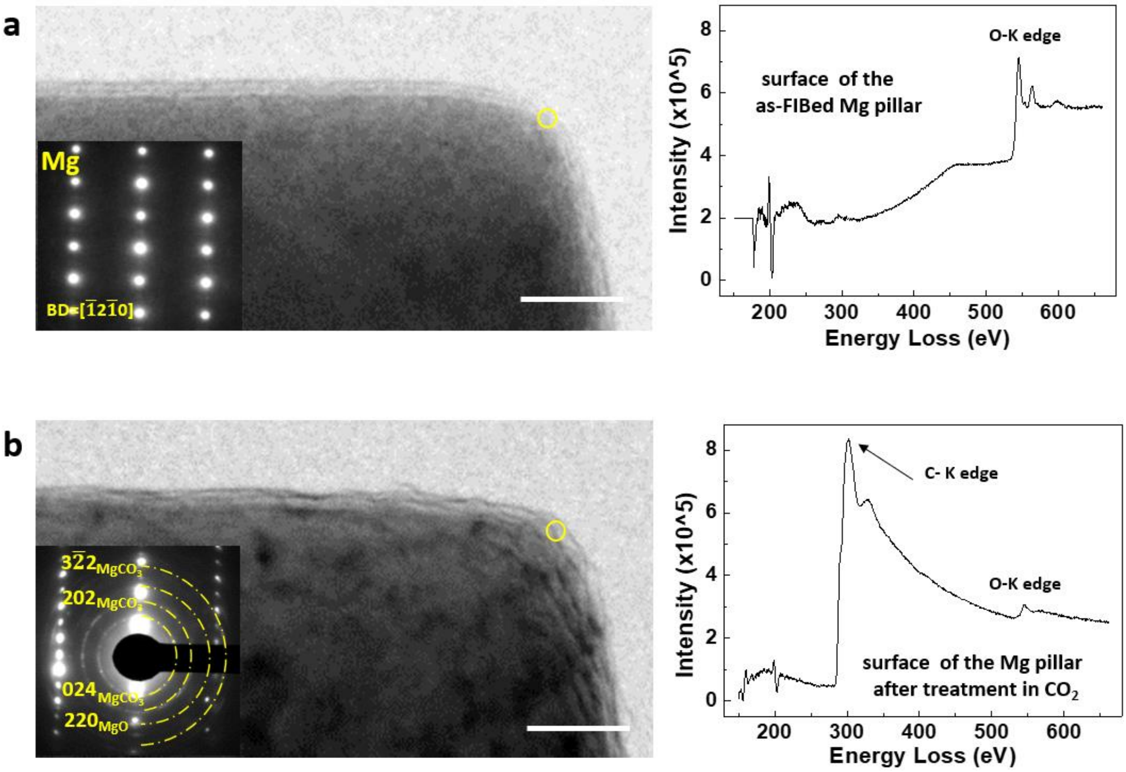

Supplementary Figure 5 | TEM characterization and EELS analysis of the Mg pillar before and after exposure to excited CO_2. **(a)** Diffraction pattern of the as-FIBed Mg pillar with an amorphous MgO layer and the EELS spectrum of the surface zone in the yellow circle. **(b)** Extra diffraction rings from the newly-formed film after treatment in CO_2 show the existence of nanocrystalline MgO and $MgCO_3$. Obvious C-K edge can be seen in the corresponding EELS spectrum. Beam direction (BD) is [$\bar{1}2\bar{1}0$]. Scale bars, 50 nm.

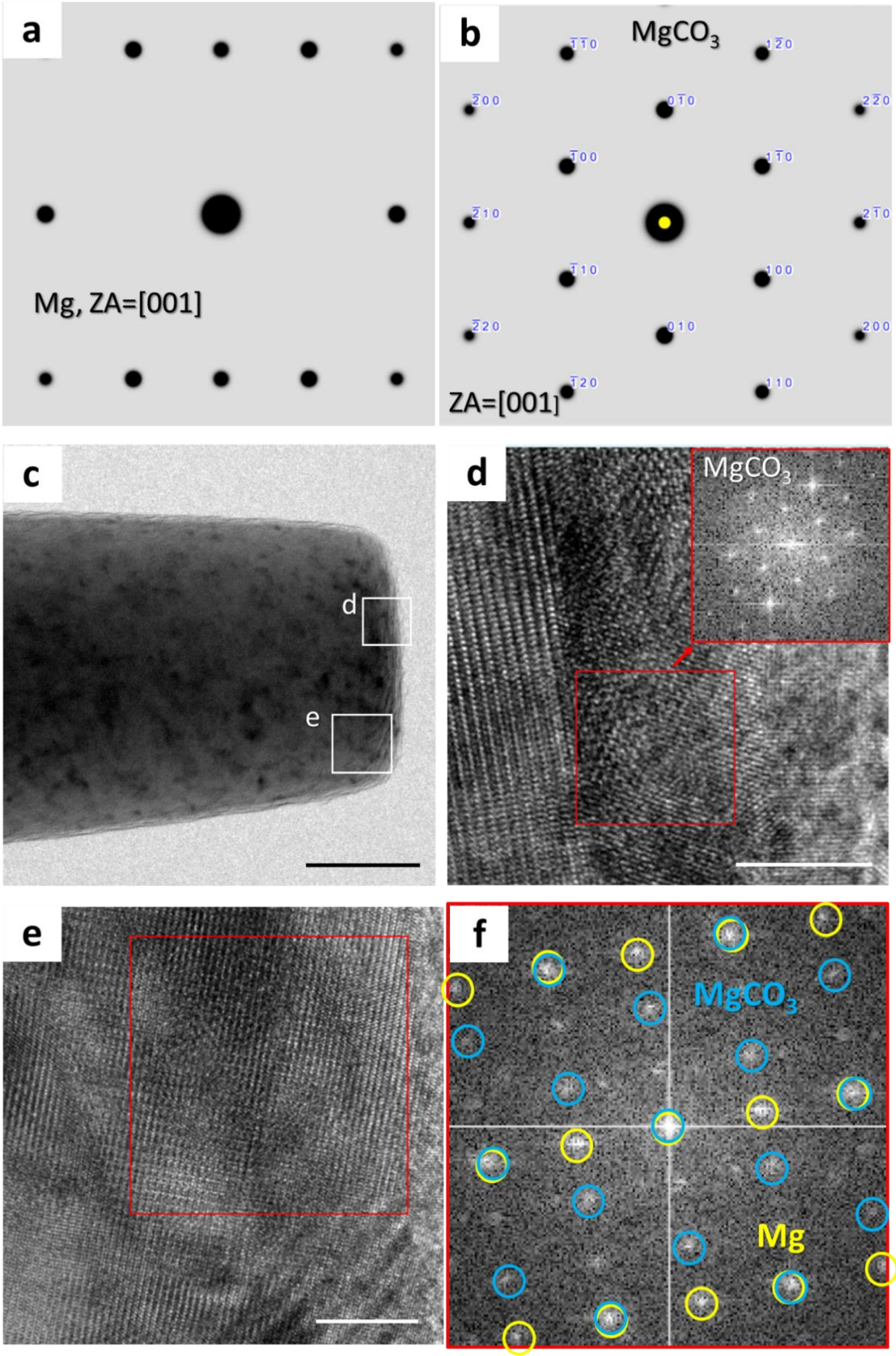

Supplementary Figure 6 | High-resolution TEM characterizations and corresponding FFT of the surface layer after treatment in excited CO_2. (a) Standard diffraction pattern of crystalline Mg with the [001] zone axis (ZA). **(b)**

Standard diffraction pattern of crystalline $MgCO_3$ with ZA=[001]. **(c)** Bright field TEM image of the Mg nanopillar after the reaction with the excited CO_2^*. **(d)** HRTEM image of the framed zone in (c), and the inset image is the corresponding FFT of the marked zone. The FFT coincides with the diffraction information of $MgCO_3$ in (b), and this indicates this small area only contains crystalline $MgCO_3$. **(e)** HRTEM image of the framed zone in (c), and **(f)** is the corresponding FFT of the marked zone. The selected area is larger, so more information in reciprocal space can be seen: the spots circled in yellow come from monocrystalline Mg substrate, and the spots circles in blue are from crystalline $MgCO_3$; other unmarked dispersed spots are due to unreacted and crystallized oxide. Scale bars: (**c**) 50 nm, (**d**), (**e**)10 nm.

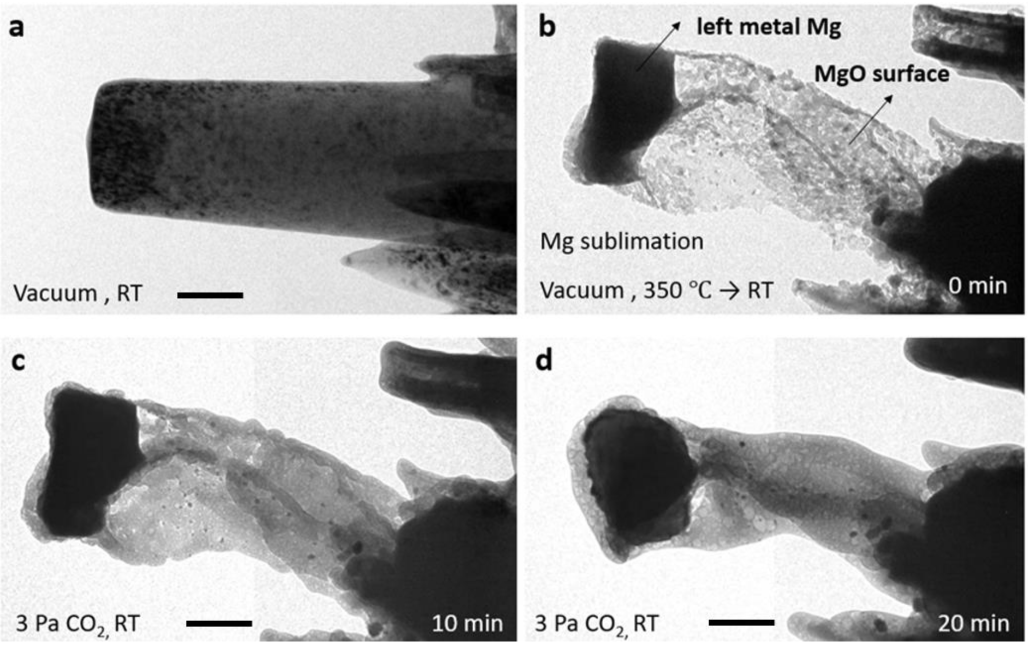

Supplementary Figure 7 | Reaction of the oxide shell of Mg pillar with CO_2 under the irradiation of e-beam. **(a)** Bright-field TEM image of the original FIBed Mg pillar. **(b)** Heated to 380°C in vacuum, most of the Mg metal sublimated away; after cooled to room temperature (RT), the MgO shell was left. **(c)** and **(d)** reaction of the MgO shell with 3 Pa CO_2 under e-beam irradiation at RT. The reaction phenomenon is almost the same as that in Fig. 1. Scale bars, 100 nm.

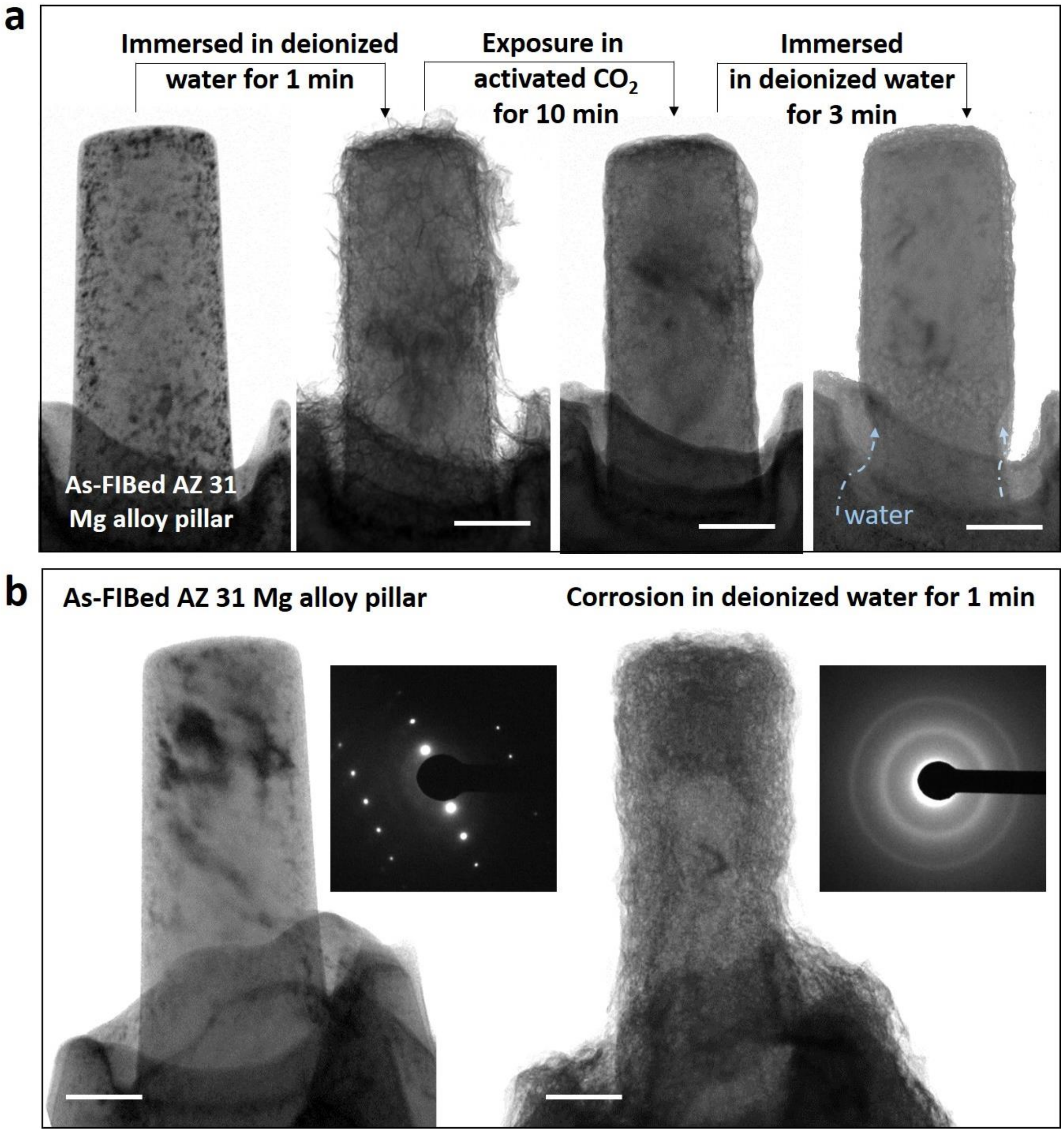

Supplementary Figure 8 | Comparison of the corrosion resistance of the as-FIBed AZ 31 Mg alloy pillars and those treated in CO_2. (a) Mitigation of corrosion on AZ 31 Mg alloy via reaction of the pre-corrosion product on surface with excited CO_2. After immersion in deionized water for 1 minute, the homogeneous corrosion product-fluffy $Mg(OH)_2$ (MgO) film grew on the FIB-fabricated pillar's surface. After exposed in the CO_2 atmosphere for 10 minutes, the formed fluid-like $MgCO_3$ covered the pillar. After immersion in deionized water for 3 min again, most of the pillar kept undamaged except for the bottom part which was not protected. Water

attacked the metal from the interior of pillar's bottom. **(b)** For comparison, the untreated AZ 31 Mg alloy pillar suffered very serious corrosion after immersion in water for only 1 minute. Scale bars, 100 nm.

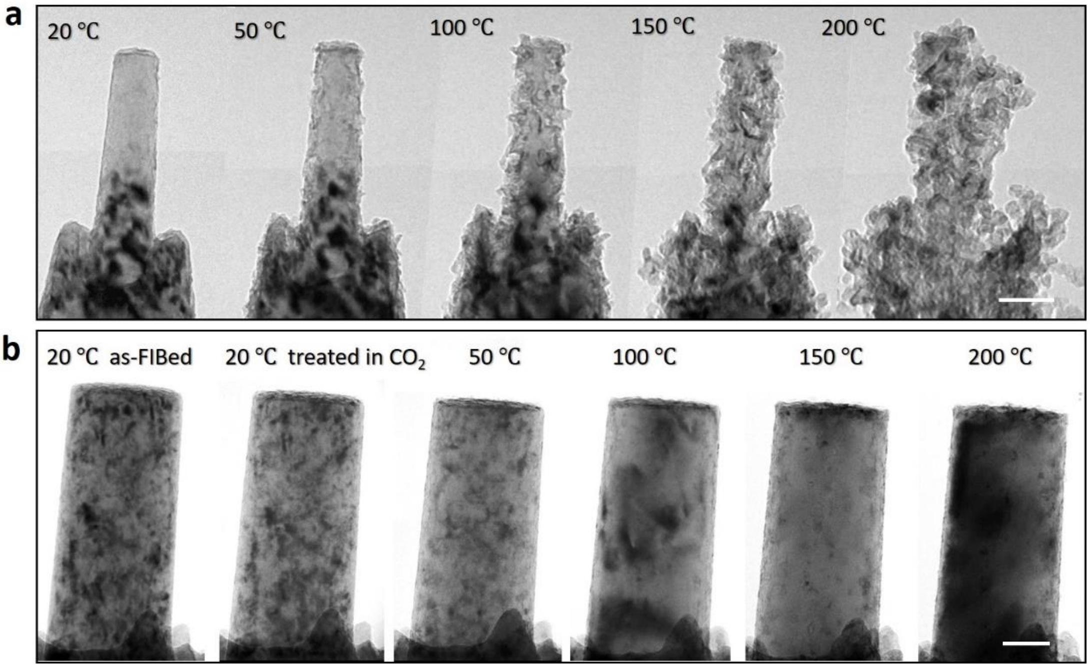

Supplementary Figure 9 | Comparison of the high temperature oxidation of the Mg pillars with and without $MgCO_3$ protective film. (a) Real-time oxidation process of the as-FIBed pure Mg nanopillar with the increasing temperature from 20°C to 200°C in 2 Pa O_2 gas. **(b)** Real-time heating process of the Mg nanopillar with $MgCO_3$ protective film with the increasing temperature from 20°C to 200°C in 2 Pa O_2. Scale bars: (**a**) 100 nm, (**b**) 50 nm.

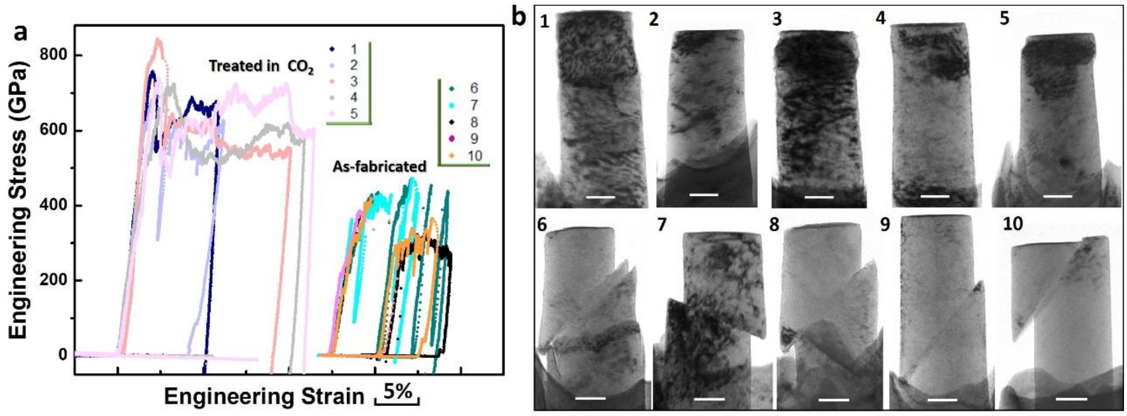

Supplementary Figure 10 | Comparison of the mechanical behaviors of the as-FIBed and the treated Mg pillars. (a) Compressive engineering stress-strain curves for the Mg pillars with protective film (number 1,2,3,4,5) and the as-fabricated Mg pillars (number 6,7,8,9,10). The ten Mg pillars are single crystalline with different orientation. And the compressive directions from 1 to 10 is [$10\bar{1}2$], [$\bar{1}100$], [$\bar{1}100$], [$23\overline{16}\bar{7}3$], [$5\bar{1}\bar{4}0$], [$\bar{1}100$], [$\bar{1}100$], [$70\bar{7}9$], [$70\bar{7}9$], and [$70\bar{7}9$], respectively. The ten pillars are corresponding to what in Figure 5c. **(b)** The morphologies of the ten compressed Mg pillars. The untreated sample showed obvious shearing steps on the surfaces after compression, and the pillars with $MgCO_3$ film had a homogeneous deformation morphology with significant dislocation storage observed inside the pillars. Scale bars, 100 nm.

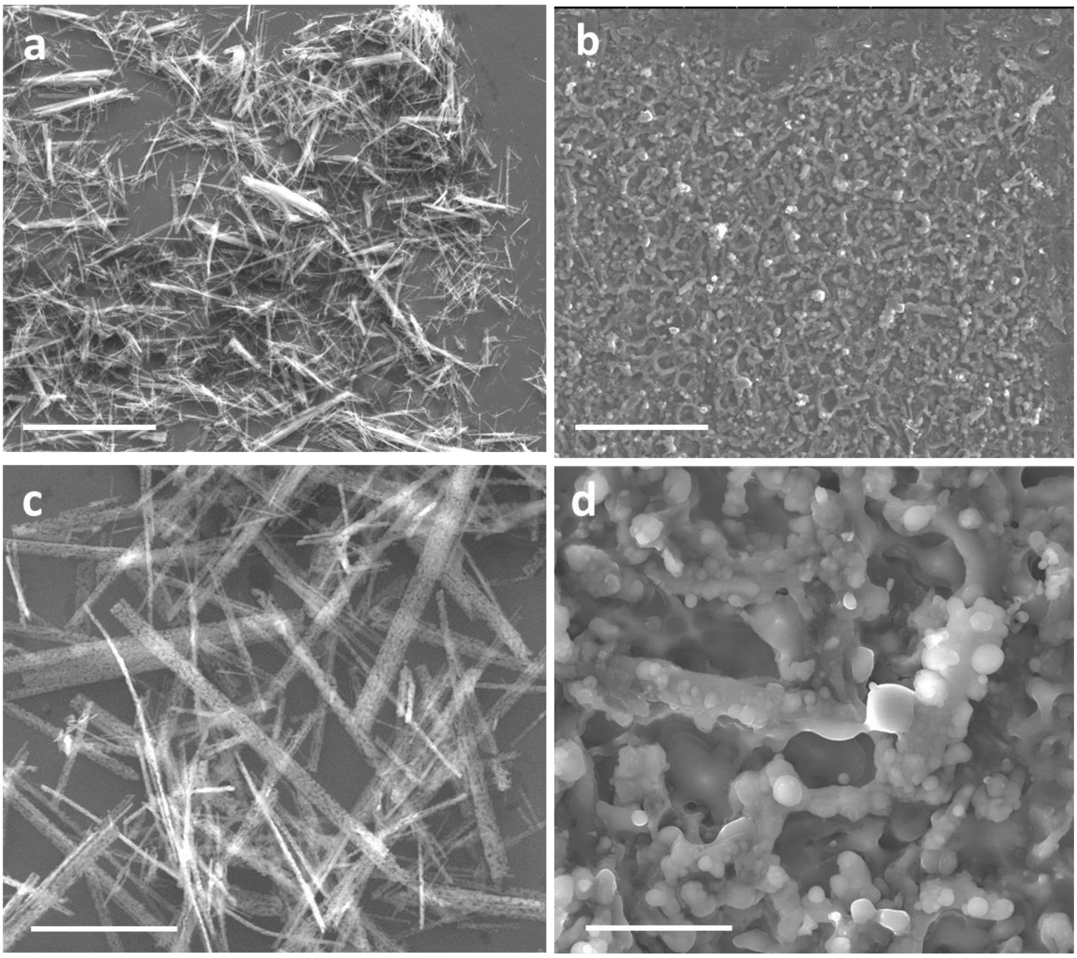

Supplementary Figure 11 | Reaction of the MgO nanorods dispersed on Si wafers with low temperature CO_2 plasma and formation of the fluid-like $MgCO_3$ similar with what produced in E-TEM. (a) Overall SEM image of the MgO nanorods (synthesized by hydrothermal method) dispersed on the Si wafer. **(b)** SEM image of the MgO rods in (a) after reaction in CO_2 plasma for 1 hour (200 Pa CO_2 atmosphere). Magnified SEM image of the MgO rods before **(c)** and after **(d)** treatment in CO_2 plasma. The final production shows the fluid-like morphology, like that formed in E-TEM experiments. The generated $MgCO_3$ can flow slowly and spread on the Si wafer uniformly. Scale bars: (**a**), (**b**) 50 μm, (**c**), (**d**)10 μm.

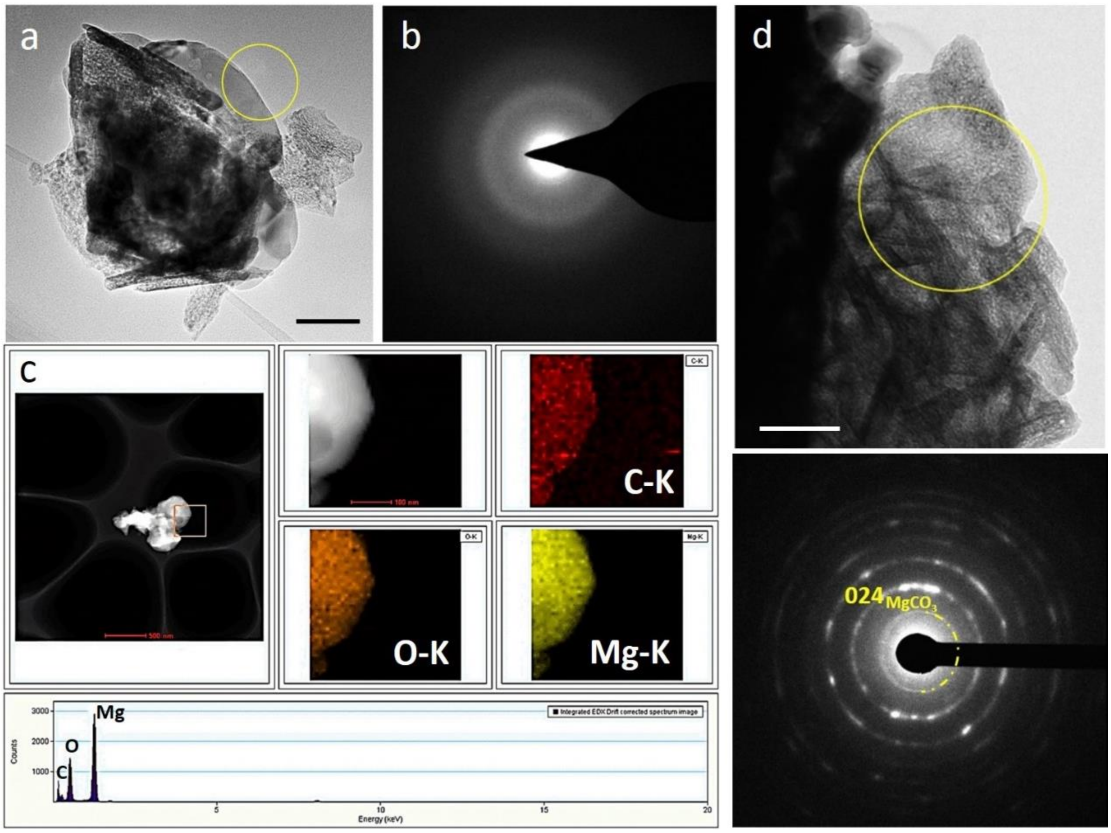

Supplementary Figure 12 | TEM characterization and element analysis of the MgO powders after plasma treatment. **(a)** TEM image of the aggregated MgO flakes after reaction with CO_2 plasma for 30 minutes (200 Pa CO_2). The reaction product was amorphous, as evidenced by the selected area diffraction halo in **(b). (c)** Elements analysis of the framed region. Energy Dispersive Spectrometer (EDS) mapping show the existence of Mg, O and C, further proving that the reaction product is $MgCO_3$. **(d)** TEM image of the aggregated MgO flakes after reaction with CO_2 plasma for 30 minutes (20 Pa CO_2). Different from what in (a), the formed $MgCO_3$ is nanocrystalline (as marked in the SAED, 024 is the characteristic diffraction ring of $MgCO_3$). Presumably, the lower CO_2 pressure decreases the reaction rate, which can favor the formation of nanocrystalline $MgCO_3$. Scale bars: (**a**) 50 nm, (**d**)100 nm.

Supplementary Note 1

Treatment of the corroded bulk pure Mg in CO_2 plasma and potentiodynamic polarization measurements

Firstly, we immersed the bulk pure Mg (99.95 wt.%) samples in deionized water for 24 hours at room temperature (25 °C) to pre-corrode the surface and obtain fluffy $Mg(OH)_2$ & MgO. After immersion, this film was found to contain a top layer of crystalline MgO embedded in an amorphous $Mg(OH)_2$ matrix[1] with an overall plate-like morphology (Supplementary Figure 13b). And then, the immersed samples were put inside a lab-made glow discharge chamber to react with CO_2 plasma for 1 hour. The corroded surface with plate-like morphology was carbonated resulting in a relatively flat and dense $MgCO_3$ surface (Supplementary Figure 13c). Anti-corrosion properties of the formed $MgCO_3$ protective films were subsequently evaluated by using potentiodynamic polarization measurements in 3.5% NaCl solution. After immersion in deionized water for 24 h and treatment in CO_2 plasma for 1 h, the sample's corrosion potential E_{corr} increased slightly and corrosion current density i_{corr} (the corrosion rate) obviously decreased, by comparing with the pristine and only immersed Mg samples (see polarization curves in Supplementary Figure 13e and Supplementary Table 1). However, from the SEM characterization of the sample after electrochemical test, we can see the top surface $MgCO_3$ film was corroded, and the plate-like MgO and $Mg(OH)_2$ underneath was exposed (Supplementary Figure 13c), which suggested that the formed $MgCO_3$ in 1-hour plasma treatment is too thin to protect the substrate from electrochemical corrosion. Therefore, we further increased the plasma treatment time to 2 hours after immersion in deionized water for 24 hours so as to form a thicker enough $MgCO_3$ protective film. 2-hours plasma treatment made $E_{\text{corr vs.SCE}}$ of Mg samples increase from -1.58 V to -1 V~ -0.73 V, and i_{corr} decrease by two orders of magnitude; after electrochemical tests, surfaces of samples kept intact and no obvious cracks on them were detected in SEM (Supplementary Figure 13d). Table S1 compares the anti-corrosion properties of $MgCO_3$ with other protective coatings formed by anodization or micro-arc oxidation. We can see that anti-corrosion effects of $MgCO_3$

are better than the anodized coating, and even comparable with the composite coatings obtained by micro-arc oxidations.

These potentiodynamic polarization tests have proved the superior corrosion resistance of bulk Mg samples with protective $MgCO_3$ film in aqueous environment. Certainly, in order to obtain the optimized anti-corrosion properties of $MgCO_3$, more experiments should be done to obtain the optimal immersion time and plasma treatment time.

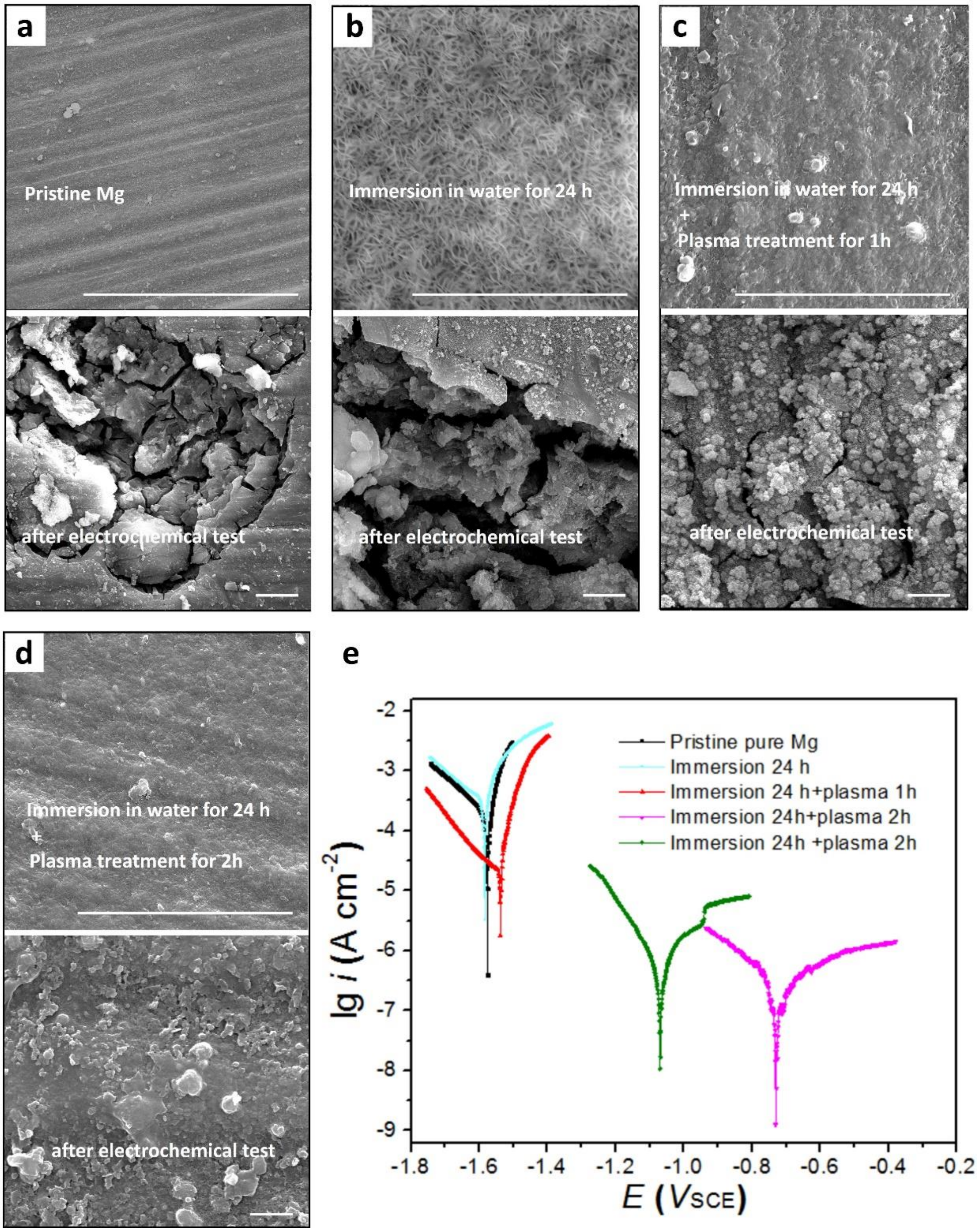

Supplementary Figure 13 | Anti-corrosion effects of $MgCO_3$ protective film on bulk Mg. Typical SEM images of the Mg samples before and after potentiodynamic polarization tests: **(a)** pristine pure Mg sample without any treatments; **(b)** pure Mg sample immersed in deionized water for 24 h; **(c)** the pure Mg sample immersed in deionized water for 24 h and then treated in CO_2 plasma for 1 h; **(d)** pure Mg sample immersed in deionized water for 24 h and then treated in CO_2 plasma for 2 h. **(e)** corresponding potentiodynamic polarization curves of above-mentioned Mg samples. Scale bars, 5 μm.

Supplementary Table 1

The results of potentiodynamic corrosion tests in 3.5 wt.% NaCl solution and comparisons with anodizing and micro-arc treatment.

Samples	E_{coor} (V_{SCE})	i_{coor} (μA/cm^2)
**Pristine Mg**	**-1.58**	**196.64**
**Immersion 24h**	**-1.59**	**251.28**
**Immersion 24h+plasma 1h**	**-1.54**	**19.52**
**Immersion 24h+plasma 2h**	**-1.06**	**0.31**
**Immersion 24h+plasma 2h**	**-0.73**	**0.22**
Anodizing coating[2]	-1.48	27
Composite coating by micro-arc oxidation[3]	-1.49	0.69
Micro-arc oxidation coating[4]	-1.69	0.17

Supplementary References

1. Nordlien, J., Ono, S., Masuko, N. & Nisancioglu, K. A TEM investigation of naturally formed oxide films on pure magnesium. *Corros. Sci.***39**, 1397-1414 (1997).
2. Xue, D., Yun, Y., Schulz, M. J. & Shanov, V. Corrosion protection of biodegradable magnesium implants using anodization. *Mater. Sci. Eng. C* **31**, 215-223 (2011).
3. Mu, W. & Han, Y. Characterization and properties of the MgF_2/ZrO_2 composite coatings on magnesium prepared by micro-arc oxidation. *Surf. Coat. Tech.* **202**, 4278-4284 (2008).
4. Zhao, L., Cui, C., Wang, Q. & Bu, S. Growth characteristics and corrosion resistance of micro-arc oxidation coating on pure magnesium for biomedical applications. *Corros. Sci.* **52**, 2228-2234 (2010).
