# Supplementary material for: Turning a native or corroded Mg alloy surface into an anti-corrosion coating in excited CO2
Source: Nat Commun. 2018 Oct 3;9:4058. doi: 10.1038/s41467-018-06433-5 (PMC6170486; doi:10.1038/s41467-018-06433-5)
Supplement: Supplementary file 3 — Description of Additional Supplementary Files [file 41467_2018_6433_MOESM3_ESM.pdf]

Description of Additional Supplementary Files

File Name: Supplementary Movie 1
Description: An in situ TEM movie showing the reaction of the MgO nanocrystals with excited CO_2 under electron beam. The movie was recorded at 5 frames/second and played at 50× speed. Snapshots of this movie are shown in Figure 2.

File Name: Supplementary Movie 2
Description: Real-time record of heating and e-beam irradiation effects on the reaction of $MgO+CO_2 \rightarrow MgCO_3$. This movie unambitiously illustrates that it is the e-beam ionization rather than its heating effect that activates this reaction. The movie was recorded at 5 frames/second and played at 50× speed. Snapshots of this movie are shown in Figure S2.

File Name: Supplementary Movie 3
Description: In situ TEM movie shows the reaction process of the brushy corrosion products (pre-existing on surface) with excited CO_2. The movie was recorded at 5 frames/second, and played at 50× speed. Snapshots of this movie are shown in Figure 4 a,b,c.
